# Birthing Centers Staffed by Skilled Birth Attendants: Can They Be Effective … *at Scale*?

**DOI:** 10.9745/GHSP-D-16-00063

**Published:** 2016-03-25

## Abstract

Peripheral-level birthing centers *may* be appropriate and effective in some circumstances *if* crucial systems requirements can be met. But promising models don’t necessarily scale well, so policy makers and program managers need to consider what requirements can and cannot be met feasibly at scale. Apparently successful components of the birthing center model, such as engagement of traditional birth attendants and use of frontline staff who speak the local language, appear conducive to use in other similar settings.

See related article by Stollak.

In this issue of *Global Health: Science and Practice*, Stollak et al.^1^ report a positive experience with maternal-newborn services for remote, primarily indigenous communities in Guatemala. The work was done by an NGO and included an important focus on community outreach and cultural sensitivity. Services were made more accessible by establishing birthing centers (*Casas Maternas*) in communities where such services hadn’t previously been available. They were staffed by skilled birth attendants (SBAs)—locally hired auxiliary nurses—who spoke the local language. The project also cultivated relationships with traditional birth attendants, who were made welcome to support women giving birth in the *Casas Maternas*. In addition, the project facilitated reliable transfer of complicated cases to higher-level care.

This case raises 2 important issues, one specific to maternal-newborn care and the other more broadly relevant to generalizability or transferability from small scale to large.

## How Effective Are SBAs in This Particular Birthing Center Model?

Well into the 1990s, to the extent that the global health community gave attention to safe motherhood, the dominant model assumed that most births would take place at home without the assistance of an appropriately skilled professional. However, by the time of the 2006 *Lancet* Maternal Survival series,^2^ with the goal of ensuring as high as possible coverage of “skilled birth attendance,” the model of peripheral-level, midwife-staffed birthing centers had gained currency. Over the past decade, as various countries have made efforts to implement such services, doubts have emerged about the effectiveness of the peripheral-level birthing center model in reducing risk of death. To provide effective labor and delivery care for a population, clearly certain conditions need to be met. Unfortunately, if service providers in such settings are inadequately equipped to manage complications or if robust provision for timely transfer to higher-level care is lacking (which has commonly been the case for these services), it is hardly realistic to expect significantly improved outcomes.^3^

But, alternatively, if such crucial requirements *can* be met, this model of provision of care may be an appropriate part of the mix of services in some settings. It certainly is possible to ensure important aspects of routine, preventive care at this level, e.g., use of uterotonic drugs during the third stage of labor. Also, in principle health workers at this level can provide clean delivery and good thermal care of the newborn. Certainly, auxiliary nurses/midwives can be enabled to reliably and competently manage non-breathing newborns, at least to the point of bag-and-mask resuscitation. And with good coordination with higher-level facilities (facilitated by the now-widespread use of mobile phones) and robust provision for transportation, timely referrals can be made for complications that exceed the capabilities of the SBAs, and this care can include pre-referral stabilization and initiation of treatment, e.g., for eclampsia. But for each of these functions there are corresponding systems requirements: supply chain, infrastructure, staffing, equipment, transport and communication systems, etc. In the situation described in the Stollak paper,^1^ although the numbers are too small to ascertain mortality impact, it may well be that the conditions needed for effectiveness of a peripheral birthing center model have been met.

The important point here is that for *any* service delivery model, including peripheral-level birthing centers, it’s not a simple matter of *whether* a model does or doesn’t work; the key question is—in the particular setting—*can the conditions required for effectiveness of an intervention or service delivery strategy be met?*

This brings us to the second important issue arising from Stollak and colleagues …

## Perils of Cookie-Cutter “Evidence-Based” Models

The field of global health suffers from a tendency to search for models that can be universally recommended. As long ago as the 1970s and earlier, inspiring examples of primary health care efforts implemented in remote areas and accomplishing major reductions in mortality prompted calls for replication at scale under government health services. Such overly simplistic response to evidence from what should have been seen as no more than proofs of concept—without careful consideration of what was required for effectiveness in varying contexts—resulted in widespread uptake and large-scale implementation of community health worker programs that in many cases were eventually found to perform poorly.^4^ The same pattern of concluding—on the basis of relatively intensively supported, small-scale experiences implemented by an NGO or research group—that similar impact can be reproduced at scale under government health services remains extremely widespread in global public health (e.g., Paul 2016^5^).

## Drawing Out Key Lessons: Avoiding Mechanical Replication

One possible conclusion arising from an experience such as described in the paper by Stollak and colleagues^1^ could be, “Based on this success, now the government should implement this model at scale.” Rather, we believe another response is needed; we should be asking: “What lessons can be drawn that can be applied for broader benefit, beyond this particular setting?” As described by Stollak et al., those involved in implementing this work have been actively discussing its implications with counterparts in government and the broader NGO community in Guatemala, and certain key features of the *Casa Materna* experience have been identified that point to needed changes in how the government approaches maternal-newborn health in remote indigenous communities.

A common pattern seen in other settings is for government to put in place a pale imitation of a successful (intensively supportive demonstration) model, a dysfunctional pattern that has been characterized as “isomorphic mimicry.”^6^ In this particular instance, the government of Guatemala is perhaps moving along a more promising track. It has identified certain key aspects of how the *Casa Materna* service has been delivered, which seem to be both important for effectiveness and feasible for government services, notably:

- Upgrading existing health posts serving such communities to include 24/7 labor and delivery care
- Ensuring that traditional birth attendants are welcome to accompany women coming to these centers
- Ensuring availability of auxiliary nurses who speak the local language
- Setting up local committees to provide for better support and accountability

What to take home from this? First, regardless of whether or not the model we’re implementing is globally recommended, we need to rigorously check to see—in the particular settings where we work—if it is actually producing its intended benefit. Are our “skilled birth attendants” actually effective in saving lives or reducing morbidity? Second, promising models can’t simply be replicated at scale; drawing lessons from such early positive experiences (implemented under rather special circumstances), we are then faced with the challenge of determining how the conditions needed for effectiveness can be met within the real-world constraints of health systems operating at large scale. *–Global Health: Science and Practice*

## References

[1] Stollak I Valdez M Rivas K Perry H Casas Maternas in the rural highlands of Guatemala: a mixed-methods case study of the introduction and utilization of birthing facilities by an indigenous population. Glob Health Sci Pract. 2016;4(1):114–131. 10.9745/GHSP-D-15-00266 27016548 PMC4807753

[2] Koblinsky M Matthews Z Hussein J Mavalankar D Mridha MK Anwar I. Going to scale with professional skilled care. Lancet. 2006;368(9544): 1377–1386. 10.1016/S0140-6736(06)69382-3. 17046470

[3] Morgan A Jimenez Soto E Bhandari G Kermode M. Provider perspectives on the enabling environment required for skilled birth attendance: a qualitative study in western Nepal. Trop Med Int Health. 2014 Dec;19(12): 1457–65. 10.1111/tmi.12390 . 25252172

[4] Walt G CHWs: are national programmes in crisis? Health Policy Plan. 1988;3(1): 1–21. 10.1093/heapol/3.1.1

[5] Paul VK. Participatory women's groups: time for integration into programmes. Lancet Glob Health. 2016 Feb;4(2): e74–5. 10.1016/S2214-109X(16)00010-3. 26823216

[6] Andrews M Pritchett L Woolcock M Escaping capability traps through problem-driven iterative adaptation (PDIA). CGD Working Paper 299. Washington (DC): Center for Global Development; 2012 Available from: http://www.cgdev.org/content/publications/detail/1426292

